# Incidence and outcomes of post-operative endophthalmitis following elective phacoemulsification cataract surgery, between 2015 and 2022

**DOI:** 10.1038/s41433-024-03281-0

**Published:** 2024-07-27

**Authors:** Magdalene Y. L. Ting, Giulio Pocobelli, Diana M. Butu, Nakul Mandal, Luke Nicholson, Sharmina R. Khan

**Affiliations:** 1 Moorfields Eye Hospital NHS Foundation Trust, 162 City Road, London, EC1V 2PD UK; 2 Ophthalmology Unit, Department of Experimental Medicine and Surgery, University of Rome “Tor Vergata”, Via Cracovia, 50, Rome, Italy; 3 Institute of Ophthalmology, University College London, 11-43 Bath Street, London, EC1V 9EL UK

**Keywords:** Epidemiology, Microbiology, Eye diseases, Prognosis

## Abstract

**Background:**

Cataract surgery remains the commonest ophthalmic surgical procedure in the UK. Post-operative endophthalmitis (POE) is a sight-threatening complication. This study presents the incidence and outcomes of POE within Moorfields Eye Hospital NHS Foundation Trust (MEH) in London, UK.

**Methods:**

We conducted a retrospective, tertiary-centre study of the incidence and outcomes of POE following elective phacoemulsification cataract surgery between 2015 and 2022.

**Results:**

We report a rate of 18 out of 154 826 (0.012%) cases of POE within seven weeks of cataract surgery. The age range was 43-87 years (mean age 67.1 years), and the male-to-female ratio was 1:1. Two cases were associated with intraoperative vitreous loss, one with anterior capsular tear extending to posterior capsular tear and the other with posterior capsular tear and dropped fragment. Two cases had a concomitant intravitreal injection, one of Triamcinolone Acetonide and the other of Dexamethasone implant (Ozurdex®), to manage chronic uveitic macular oedema. The majority of samples (89.9%) resulted in Gram-positive species. A final best corrected visual acuity (BCVA) of Snellen 6/36 or better was achieved in 12/18 (66.67%). The time from cataract surgery to suspicion of POE ranged from 3 to 44 days (mean 15.8 days). Eight cases (44.4%) required pars plana vitrectomy (PPV).

**Conclusions:**

We demonstrate an incidence of POE of 0.012% following phacoemulsification at MEH from January 2015 to December 2022. Such results are a testament to high-quality surgery, training supervision and post-operative care whilst adhering to local and international standards.

## Introduction

Cataract surgery remains the commonest surgical procedure performed by the National Health Service (NHS), with an estimated 499 000 operations taking place in England and Wales in 20212022 [1].

Although relatively rare, post-operative endophthalmitis (POE) remains one of the most severe, sight threatening complications following cataract surgery. The Royal College of Ophthalmologists Cataract National Ophthalmology Database (NOD) provides national United Kingdom (UK) audit data. The Cataract NOD dataset showed that from 2006 to 2010, the national rate of POE within three months of cataract surgery was 0.03% (43/145 868 cases) [2].

This study aims to identify the POE rates after cataract surgery across Moorfields Eye Hospital NHS Foundation Trust (MEH), to specify the associated microbial growth, and to assess final visual outcomes.

## Subjects and methods

Electronic medical record data (Openeyes® and Medisoft®) were retrospectively reviewed from MEH over eight years from 2015 to 2022 to identify POE cases after elective cataract surgery. The tenets of the Declaration of Helsinki were followed.

The current protocol for elective phacoemulsification at MEH does not include preoperative antibiotic prophylaxis. However, before cataract surgery, preoperative g. Povidone Iodine (PVP) 5%w/v is instilled into the conjunctival sac for greater than 3 min. Intracameral Cefuroxime 1 mg/0.1 ml (Aprokam®) is administered at the end of the surgery, provided no contraindications exist, such as a positive history of anaphylactic reaction to beta-lactam antibiotics in which case subconjunctival Gentamicin is used.

MEH has a detailed guideline on emergent management of cases of acute post-phacoemulsification presumed bacterial endophthalmitis. In addition to clinical examination, the initial assessment involves performing a B-scan to document the presence of vitritis and rule out a retinal detachment. The B-scan is repeated after 24–48 h. Blood tests include full blood count (FBC), eritrosedimentation rate (ESR), C-reactive protein (CRP), urea and electrolytes (U&E) and liver function test (LFT). The guideline recommends urgent topical steroids at high frequency, aqueous humour and vitreous sampling under sterile conditions, and intravitreal antibiotic administration of Vancomycin (2 mg/0.1 ml) and Amikacin (0.4 mg/0.1 ml). All patients have aqueous and vitreous sampling under subtenons anaesthesia, prioritising the vitreous sample in a sterile environment in the emergency theatre. The culture media used include Chocolate agar, Sabouraud’s agar and brain heart infusion enrichment broth. The samples are also sent for polymerase chain reaction (PCR) analysis. Following the intravitreal therapy, patients are continued on high-frequency g. Dexamethasone 0.1% hourly, g. Chloramphenicol 0.5% four times a day, g. Cyclopentolate 1% three times a day and per os (PO) Moxifloxacin 400 mg once a day for ten days. Oral analgesia is also prescribed. At the 48-h review, oral Prednisolone 1 mg/kg (maximum 80 mg/day) may be started if there are clinically evident signs of improvement. The oral steroids can be weaned by 10 mg every five days if the inflammatory signs resolve. If the clinical condition is not improving or there is a deterioration, a repeat intravitreal sampling and an antibiotic injection is performed under the uveitis specialist clinic directorate; alternatively, the patient can be referred to the vitreoretinal team to consider vitrectomy.

## Results

A total of 154 826 elective phacoemulsification-cataract surgery were performed at MEH between 2015 and 2022: in this cohort were, 46.95% carried out by consultants and 53.05% by trainee surgeons, and we recorded 18 cases of POE (0.012%) as per Table 1. As detailed in Table 2, the patient’s mean age was 67.1 (range 43-87 years). The male-to-female ratio was 1:1. Sixteen cases (88.9%) resulted in endophthalmitis following uncomplicated cataract operations. Two of the remaining cases (11.1%) were associated with intraoperative complications: anterior capsule tear with vitreous loss and posterior capsular rupture with dropped lens material; the first requiring anterior vitrectomy only and the latter requiring primary anterior vitrectomy and secondary fragmatome and pars plana vitrectomy.

**Table 1:** Total numbers of cataract cases per year and respective endophthalmitis rates.

Year	Total cataracts	POE	POE rate	POE rate
2015	19332	2	0.01	0.010%
2016	19914	1	0.005	0.005%
2017	19749	1	0.005	0.005%
2018	20840	5	0.024	0.024%
2019	22070	5	0.023	0.023%
2020	11770	0	0	0.000%
2021	16588	2	0.013	0.012%
2022	24563	2	0.01%	0.008%
Total	154826	18	0.012%	0.012%

**Table 2:** Demographics and factors associated with POE.

No	Age	Sex	Site of cataract surgery	Side	Surgeon grade	Main incision length (mm)	Immunodeficient/Increased infection risk	Previous IVT or Vitrectomy	Intraoperative complication	10/0 nylon post complication	Intracameral Cefuroxime	Intravitreal antibiotics	Oral Moxifloxacin	Oral Prednisolone and dose	Time to final follow up (days)	Presenting or earliest recorded VA (Snellen)	Final VA (Snellen)	Time of Dx from cataract operation (days)	Post-Dx Vitrectomy	Time to vitrectomy (days)	Microbial growth
1	57	M	City Road	Left	Consultant	9.9	No	Vity	Posterior capsular tear, dropped nucleus, vitreous loss	Yes	Yes	Yes	No	No	126	6/12	6/9	11	No	N/A	No growth
2	78	M	Whittington	Left	Consultant	2.4	Yes (diabetes, vasculitis on Prednisolone)	No	No	N/A	Yes	Yes	Yes	Yes, 50 mg	219	6/24	6/9	8	Yes	103	*Pseudomonas aeruginosa*
3	49	F	City Road	Left	Fellow	2.4	No	No	No	N/A	No (subconj Cefuroxime and Betnesol 4 mg instead)	Yes	Yes	Yes, 60 mg	329	CF	6/18	3	No	N/A	*Staph. Epidermidis*
4	73	F	Queen Mary’s	Right	Consultant	2.4	Yes (Rheumatoid arthritis, colon carcinoma)	No	No	N/A	Yes	Yes	Yes	Yes, 60 mg	415	HM	6/5	6	No	N/A	*Staph. epidermidis*
5	82	F	Queen Mary’s	Right	Registrar	2.4	Yes (diabetes, endometrial carcinoma)	No	No	N/A	Yes	Yes	Yes	Yes, 75 mg	502	HM	HM	5	Yes	7	*Staph. epidermidis*
6	87	F	St Anthony’s	Left	Consultant	2.4	No	No	No	N/A	Yes	Yes	Yes	No	360	6/36 (on the day)	6/12	8	No	N/A	*No growth*
7	56	M	City Road	Right	Consultant	2.4	No	No	No	N/A	Yes	Yes	Yes	No	1083	HM (on the day)	6/6	22	No	N/A	*No growth*
8	58	M	St Ann’s	Left	Associate specialist	2.4	No	No	No	N/A	Yes	Yes	Yes	Yes, 60 mg	464	HM	6/6	6	No	N/A	*Strep. sanguinis*
9	76	M	Ealing	Right	Associate specialist	2.4	Yes (diabetes)	No	No	N/A	Yes	Yes	Yes	No	223	6/18	6/12	23	No	N/A	*Micrococcus species*
10	83	F	Ealing	Left	Consultant	2.4	No	No	Anterior capsular tear, vitreous loss	Yes	Yes	Yes	Yes	Yes, 60 mg	778	PL	PL	19	Yes	4	*Strep. sanguinis*
11	66	F	Ealing	Right	Consultant	2.4	Yes (diabetes)	No	No	N/A	Yes	Yes	Yes	Yes, 30 mg	77	CF (on the day)	2/60	40	No	N/A	*No growth*
12	54	M	Potters Bar	Right	Consultant	2.4	No	No	No	N/A	Yes	Yes	Yes	Yes, 50 mg	713	6/18	6/9	10	No	N/A	*Staph. warneri*
13	84	F	City Road	Right	Associate specialist	2.4	No	No	No	N/A	Yes	Yes	Yes	No	247	6/18	6/9	44	No	N/A	*No growth*
14	57	F	St George’s	Right	Consultant	2.4	No	No	No	N/A	Yes	Yes	Yes	Yes, 60 mg	687	CF (on the day)	HM	3	Yes	18	*No specimen obtained*
15	70	M	St Ann’s	Right	Fellow	2.4	No	Vity	No	N/A	Yes	Yes	Yes	Yes, 60 mg	439	HM	6/6	41	Yes	1	*No growth*
16	43	F	City Road	Right (combined paco+iol+ivt triacense)	Consultant	2.4	Yes (prednisolone 40 mg to manage CAU)	No	No	N/A	Yes	Yes	Yes	Yes, 40 mg	344	HM	6/60	3	Yes	3	*Staph. epidermidis*
17	69	M	St George’s	Right	Registrar	2.4	No	No	No	N/A	Yes	Yes	Yes	No	247	6/36	6/6	9	Yes	11	*Staph. epidermidis*
18	71	M	St George’s	Right (combined paco+iol+ivt Ozurdex)	Consultant	2.4	Yes (diabetes, MALT)	No	No	N/A	Yes	Yes	Yes	80 mg	117	HM	NPL	24	Yes	26	*Pseudomonas Aeruginosa*

*IVT* intravitreal injection therapy, *POE* post-operative endophthalmitis, *VA* visual acuity.

Out of the 18 POEs, only 4 (22,2%) occurred after surgeries performed by trainees.

On presentation to the eye emergency department, all the cases received intravitreal Vancomycin and Amikacin. All patients (18/18) also received oral Moxifloxacin 400 mg daily for ten days. Oral Prednisolone was required in 12/18 (66.7%) at 48 h post-presentation.

Overall, 7/18 cases (38.9%) had relevant general and ocular conditions, which included an active neoplasm, rheumatoid arthritis requiring immune modifying therapy, chronic uveitis requiring steroid anti-inflammatory therapy and diabetes mellitus. 2/18 cases (11.1%) had a history of previous pars plana vitrectomy.

In 17/18 (94.4%) patients, we recorded the use of intracameral Cefuroxime (Aprokam®), and one case (0.6%) had a Penicillin allergy, so subconjunctival Gentamicin and Betnesol were administered instead. A final best corrected visual outcome of Snellen 6/36 unaided or better was achieved in 12 cases (66.7%); 9 (50%) patients obtained Snellen 6/9 or better; 6/18 (33.3%) had final visual acuity of less than 6/36 best corrected. The mean final visual acuity follow-up time was 409.4 days (range 77-1083 days). The time from cataract surgery to diagnosis and treatment of POE ranged from 3 to 44 days (mean 15.8 days). 8/18 (44.4%) cases underwent pars plana vitrectomy between 1 to 103 days from the presentation with POE (mean 21.6 days).

Samples from the aqueous humour and the vitreous cavity were collected in all cases. There was a failure to collect adequate volume from the aqueous and the vitreous in 4 (22.2%) and 2 (12.50%) cases, respectively; 5 patients (27.7%) showed no growth from both aqueous and vitreous samples.

Overall, 11/18 (61.11%) samples yielded microbial growth, which was as follows: 4 cases (22.2%) of Staphylococcus epidermidis, 3 cases (16.7%) of Streptococcus species, 2 cases of Micrococcus species (11.1%), 2 cases of Pseudomonas aeruginosa (11.1%), 1 case (5.6%) of Staphylococcus Warneri. Notably, most samples (89.9%) yielded Gram-positive species, while no fungal endophthalmitis was identified. All the positive samples from the aqueous humour produced Grampositive species and only two vitreous samples were found to be positive for a Gram-negative species (Table 3).

**Table 3:** Microbiology results.

Patient	Aqueous sample	Vitreous sample
1	No growth	No growth
2	No growth	*Pseudomonas Aeruginosa*
3	No growth	*Staphylococcus epidermidis*
4	No growth	*Staphylococcus epidermidis*
5	No growth	*Staphylococcus epidermidis*
6	No sample	No growth
7	No growth	No sample
8	*Strep.Sanguinis*	No growth
9	*Micrococcus luteus*	No growth
10	No sample	*Streptococcus sanguinis*
11	No growth	No growth
12	*Staphylococcus warneri*	No growth
13	No growth	No sample
14	No sample	No growth
15	No sample	No growth
16	No growth	*Micrococcus luteus*
17	Staphylococcus Epidermidis	*Staphylococcus Epidermidis, Streptococcus Mitis*
18	No growth	*Pseudomonas Aeruginosa*

## Discussion

We report a stable trend in endophthalmitis rate over eight years from 0.01% (2/19 332) in 2015 to 0.01% (2/24 563) in 2022. This is consistent with the current literature, which has also reported stable or decreasing trends in endophthalmitis rates following cataract surgery [3–6]. The study’s findings align with the global efforts to improve perioperative strategies, such as eyelid draping and proper use of antiseptics like g. PVP 5%w/v and the administration of intracameral antibiotics reflect a commitment to maintaining high standards of surgical care and post-operative management [7–12].

However, recent evidence from in vitro studies suggests that the current recommendations of 3 min of antisepsis prior to intraocular surgery may not be sufficient to eradicate multidrug-resistant (MDR) organisms, particularly Enterococcus Spp, from the ocular surface [13]. The current practice of prophylactic intracameral Cefuroxime is based on evidence from the landmark ESCRS Endophthalmitis study 2007 which showed a fivefold reduction in the incidence of POE in the Cefuroxime arm [14]. However, we are cognizant of side effects associated with inadvertent intracameral injection of greater than 0.1 ml of Cefuroxime include cystoid macular oedema, serous retinal detachment, and toxic anterior segment syndrome [15]. The use of intracameral Moxifloxacin 0.5 mg/0.1 ml has recently been recommended by the Royal College of Ophthalmologist as an alternative for patients with a history of anaphylaxis to Penicillin. Fourth-generation Fluoroquinolones are used extensively in the USA, partly because of the lack of pre-prepared Cefuroxime widely available in Europe. Despite no prospective trial conducted for i/c Moxifloxacin, extensive observational data supports its efficacy. In a recent metanalysis, seventeen studies included over 900,000 eyes; favouring intracameral antibiotic use during phacoemulsification cataract surgery (OR 0.20; 95% CI 0.13 to 0.32; P < 0.00001) with an average weighted postoperative endophthalmitis incidence rate with intracameral Cefuroxime, Moxifloxacin and Vancomycin were 0.0332%, 0.0153% and 0.0106% respectively [16].

Although rare at standard dosing, retinal toxic events were least associated with i/c Moxifloxacin, and higher with i/c Vancomycin. The use of intracameral Vancomycin has been progressively discouraged due to the risk of systemic drug resistance and ocular side effects such as haemorrhagic vasculitis, which can lead to severe vision loss, with standard dosage [17].

Furthermore, risk-stratified selection for Specialty Trainees and Fellows according to skill can decrease rates of surgical complications and subsequent POE [18]. Indeed, the UK Cataract National Ophthalmology Dataset (NOD) showed cataract surgery complicated by posterior capsule rupture (PCR) was associated with an approximately sevenfold increase in POE [1]. Our results do not confirm these observations, and our rates were favourable in comparison, as we measured a PCR rate of 1.19% over the study period. Only 4/18 of the POEs occurred after interventions performed by trainees. Therefore, despite evidence from other publications that support the risk of POE is higher in cases of vitreous loss, which is more frequent among trainees; in our study we did not find any clinically significant correlation between the occurrence of intraoperative complications and occurrence of POE. This is likely due to prompt management of intraoperative complications by the supervising senior surgeon [1, 18].

We employ pre-operative efforts in judicious risk stratification of patients according to a surgeon’s level of experience, which are designed towards favourable outcomes by reducing the incidence of intraoperative complications that might reflect a low POE rate. Furthermore, close supervision by senior surgeons and timely recognition of complications and appropriate management should mitigate outcomes associated with complications under trainee surgeons.

Moreover, there were no instances of previous intravitreal injection therapy (IVT) in our POE cohort, previously associated with an increased risk of intraoperative complication and subsequent development of endophthalmitis [19].

We considered the timing of PPV in patients that required this intervention and observed varying BCVA outcomes. One patient who underwent “early PPV” 1-day post-presentation of POE had BCVA 6/6 at final follow up. Similarly, a patient underwent “late PPV” after 103 days had a BCVA 6/9 at final follow-up. In contrast, the one patient underwent “early PPV” 4 days after the diagnosis of POE, had BCVA perception of light (PL) at final follow up. While these observations may suggest a potential benefit of “early PPV” in some cases, it is important to note that these results are based on a small number of cases. The complexity of the case, severity of infection, individual patient characteristics, and other treatment modalities employed contributed to the outcomes observed.

The Endophthalmitis Vitrectomy Study (EVS), published in 1995, was the first landmark study encouraging the standardisation of the management of POE. The EVS concluded that visual prognosis was linked to causative organisms and the presenting visual acuity; and recommended early pars plana vitrectomy for cases presenting with perception of light or worse [20]. A more recent retrospective study reported old age, infection by gram-negative organisms (especially Pseudomonas aeruginosa), short time between cataract operation and signs of endophthalmitis, and poor presenting visual acuity as negative visual prognostic factors [21]. Results from a recent tertiary centre retrospective case series suggest that an increase in the scope of the EVS indications for early vitrectomy could be advantageous [22]. Another research group recommended that early pars plana vitrectomy could be considered if the clinical response was poor within 48 h following the initial intravitreal antibiotic injection [23]. There remains a lack of high-quality evidence which could be gained through an up-to-date randomised controlled clinical trial on the role of early vitrectomy in managing acute POE.

A limitation of our study is the small number of cases, further observation with a larger sample size allowing for statistical analysis would be beneficial.

Nonetheless, the results suggest that the teaching hospital environment, with its focus on education and supervision, can create a conducive setting for trainees to learn and perform surgeries while maintaining patient safety and minimizing complications like POE.

There was no growth of microorganisms in some cases of post-operative endophthalmitis (POE). It is important to acknowledge the limitations of the culture method used in this study and potential false-negative results. Advances in diagnostic techniques, including RT-PCR and mass spectrometry, hold promise for improving the detection of microorganisms in endophthalmitis cases. Further research and evaluation of these methods are needed to establish their utility and feasibility in routine clinical practice.

In summary, we demonstrate an incidence of POE of 0.012% (18/154 826) following phacoemulsification cataract surgery at Moorfields Eye Hospital NHS Foundation Trust from 2015 to 2022. This low rate of POE is in concordance with reports and trends globally. Such commendable rates are indicative of high-quality surgery, close supervision of trainees and comprehensive postoperative care undertaken to the highest global standards.

## Summary

### What was known before

- Post-operative endophthalmitis (POE) remains the most serious post-operative complication following cataract surgery resulting in severe visual loss.
- Copious pre-operative g Povidone Iodine 5%w/v in the conjunctival sac and intracameral antibiotics are recommended to minimise the risk of developing POE.

### What this study adds

- We report a POE incidence of 0.012% (18/154 826) following elective phacoemulsification-cataract surgery at a high-volume tertiary centre in London, where 53.05% of cases were carried out by an Ophthalmology Specialty Trainee (OST) surgeon from 2015 to 2022.
- We report the final best corrected visual acuity of better than 6/36 in 12 out of 18 cases (66.7%); severe visual loss after POE can be limited if this condition is promptly diagnosed and treated.
- The aqueous and vitreous microbiology results in this cohort of patients are associated with a higher prevalence of Gram-positive bacteria.

## Supplementary information

Reporting Checklist

## Data Availability

Data from Tables 1, 2, 3 were obtained from the Data Warehouse Manager of the Performance and Information Department as well as from the Infection Control Team of Moorfields Eye Hospital Foundation Trust. 2015 to 2021 data are available publicly on Moorfields Eye Hospital’s Infection Control Annual Reports.
