# Correction: Incidence and outcomes of post-operative endophthalmitis following elective phacoemulsification cataract surgery, between 2015 and 2022

**DOI:** 10.1038/s41433-024-03457-8

**Published:** 2024-11-07

**Authors:** Magdalene Y. L. Ting, Giulio Pocobelli, Diana M. Butu, Nakul Mandal, Luke Nicholson, Sharmina R. Khan

**Affiliations:** 1 Moorfields Eye Hospital NHS Foundation Trust, London, UK; 2 Ophthalmology Unit, Department of Experimental Medicine and Surgery, University of Rome “Tor Vergata”, Rome, Italy; 3 Institute of Ophthalmology, University College London, London, UK

**Keywords:** Epidemiology, Microbiology, Eye diseases, Prognosis

Correction to: *Eye* 10.1038/s41433-024-03281-0, published online 27 July 2024

In Table 3, identifiable patient numbers were inadvertently included and have now been removed. The original article has been corrected.

