# Supplementary material for: Incidence and outcomes of post-operative endophthalmitis following elective phacoemulsification cataract surgery, between 2015 and 2022
Source: Eye (Lond). 2024 Jul 27;38(18):3429–33. doi: 10.1038/s41433-024-03281-0 (PMC11621521; doi:10.1038/s41433-024-03281-0)
Supplement: Supplementary file 1 — Reporting Checklist [file 41433_2024_3281_MOESM1_ESM.pdf]

Corresponding Author Name: Sharmina R. K
Manuscript Number: EYE-23-1341

Reporting Checklist

This checklist is used to ensure good reporting standards and to improve the reproducibility of published results. **Please respond completely to all questions relevant to your manuscript, indicating "N/A" if the question does not apply.** For more information, please read the journal's Guide to Authors.

■ Check here to confirm that the following information is available in the Material & Methods section:

- the **exact sample size (*n*)** for each experimental group/condition, given as a number, not a range;
- a **description of the sample collection** allowing the reader to understand whether the samples represent **technical or biological replicates** (including how many animals, litters, culture, etc.);
- a **statement of how many times the experiment shown was replicated in the laboratory**;
- **definitions of statistical methods and measures**: (For small sample sizes (n<5) descriptive statistics are not appropriate, instead plot individual data points)
  - very common tests, such as *t*-test, simple χ^2 tests, Wilcoxon and Mann-Whitney tests, can be unambiguously identified by name only, but more complex techniques should be described in the methods section;
  - are tests one-sided or two-sided?
  - are there adjustments for multiple comparisons?
  - **statistical test results**, e.g., ***P* values**;
  - definition of **'center values'** as **median or mean**;
  - definition of **error bars as s.d. or s.e.m. or c.i.**

Please ensure that the answers to the following questions are reported **in the manuscript itself.** We encourage you to include a specific subsection in the methods section for statistics, reagents and animal models. Below, provide the page number or section and paragraph number.

Statistics and general methods	Reported in section/paragraph or page #
1. How was the sample size chosen to ensure adequate power to detect a pre-specified effect size? (Give section/paragraph or page #)	Not applicable
For animal studies, include a statement about sample size estimate even if no statistical methods were used.	Not applicable
2. Describe inclusion/exclusion criteria if samples or animals were excluded from the analysis. Were the criteria pre-established? (Give section/paragraph or page #)	Not applicable
3. If a method of randomization was used to determine how samples/animals were allocated to experimental groups and processed, describe it. (Give section/paragraph or page #)	Not applicable
For animal studies, include a statement about randomization even if no randomization was used.	Not applicable

4. If the investigator was blinded to the group allocation during the experiment and/or when assessing the outcome, state the extent of blinding. (Give section/paragraph or page #)	Not applicable
For animal studies, include a statement about blinding even if no blinding was done.	Not applicable
5. For every figure, are statistical tests justified as appropriate?	Not applicable
Do the data meet the assumptions of the tests (e.g., normal distribution)?	Not applicable
Is there an estimate of variation within each group of data?	Not applicable
Is the variance similar between the groups that are being statistically compared? (Give section/paragraph or page #)	Not applicable

**Reagents**	**Reported in section/paragraph or page #**
6. Report the source of antibodies (vendor<br>7. and catalog number)	Not applicable
8. Identify the source of cell lines and report if they were recently authenticated (e.g., by STR profiling) and tested for mycoplasma contamination	Not applicable

**Animal Models**	**Reported in section/paragraph or page #**
8. Report species, strain, sex and age of animals	Not applicable
9. For experiments involving live vertebrates, include a statement of compliance with ethical regulations and identify the committee(s) approving the experiments.	Not applicable

10. We recommend consulting the ARRIVE guidelines (*PLoS Biol.* **8**(6), e1000412,2010) to ensure that other relevant aspects of animal studies are adequately reported.

**Human subjects**	**Reported in section/paragraph or page #**
11. Identify the committee(s) approving the<br>12. study protocol.	Not applicable
13. Include a statement confirming that informed consent was obtained from all subjects.	Not applicable
14. For publication of identifiable patient photos, include a statement confirming that consent to publish was obtained. This is not required for photos just of the eye which do not identify the patient.	Not applicable
15. Report the clinical trial registration number (at ClinicalTrials.gov or equivalent).	Not applicable

16. For phase II and III randomized controlled trials, please refer to the CONSORT statement and submit the CONSORT checklist with your submission.

17. For tumor marker prognostic studies, we recommend that you follow the REMARK reporting guidelines.
